# Transcervical carotid artery stenting compared to transfemoral carotid artery stenting and carotid endarterectomy: perioperative and short-term results from a single center

**DOI:** 10.3389/fcvm.2026.1743275

**Published:** 2026-06-03

**Authors:** Wenxu Jin, Haizhen Ni, Chongqing Huang, Lemen Pan, Yihui Qiu, Jingyong Huang, Guanfeng Yu

**Affiliations:** Department of Vascular Surgery, The First Affiliated Hospital of Wenzhou Medical University, Wenzhou, Zhejiang Province, China

**Keywords:** carotid artery disease, carotid artery stenosis, carotid endarterectomy (CEA), transcervical carotid artery stenting (TC-CAS), transfemoral carotid artery stenting (TF-CAS)

## Abstract

**Background:**

Carotid artery stenosis is a major cause of ischemic stroke. Various revascularization strategies exist, including Carotid Endarterectomy (CEA), Transfemoral Carotid Artery Stenting (TF-CAS), and Transcervical Carotid Artery Stenting (TC-CAS). This study aimed to compare the perioperative outcomes and six-month follow-up results among patients undergoing TC-CAS, TF-CAS, and CEA for carotid artery stenosis.

**Methods:**

A retrospective analysis was performed on 220 patients undergoing carotid revascularization from 2020 to 2024. Patients were divided into three groups: TC-CAS (50), TF-CAS (81), and CEA (89). Primary endpoints included perioperative stroke and complications; secondary endpoints assessed six-month restenosis and stroke/TIA events.

**Results:**

Perioperative stroke rates were low and comparable among all groups (TF-CAS 2.5%, TC-CAS 2.0%, CEA 1.1%; *P* = 0.802). Cranial nerve injury occurred exclusively in the CEA group. Six-month restenosis rates showed no significant difference (TF-CAS 3.7%, TC-CAS 2.0%, CEA 4.5%; *P* = 0.752), with no stroke or TIA events observed in the TC-CAS group. Hospital costs were significantly lower in the CEA group (25.0 ± 9.8 × 10^3^ CNY) compared to TF-CAS (57.6 ± 22.8 × 10^3^ CNY) and TC-CAS (56.4 ± 20.1 × 10^3^ CNY) (*P* < 0.001).

**Conclusion:**

TC-CAS is a feasible and promising alternative to TF-CAS and CEA, offering favorable perioperative safety and short-term clinical outcomes. It is particularly suitable for patients with high-risk anatomy, but longer-term studies are required to confirm sustained efficacy.

## Introduction

The global prevalence of carotid stenosis is estimated to be 1.8% in men and 1.2% in women aged 30–79 years (1). Carotid artery stenosis is one of the primary pathogenic mechanisms of ischemic stroke, accounting for approximately 15%–20% of all ischemic stroke events (2, 3).

Currently, the primary treatment strategies for carotid artery stenosis include Best Medical Therapy (BMT), Carotid Endarterectomy (CEA), and Carotid Artery Stenting (CAS). As the “gold standard” treatment, CEA has demonstrated well-established efficacy in stroke prevention supported by long-term follow-up data (4, 5). However, being an open surgical procedure, it carries risks of anesthesia-related complications such as cardiac infarction. Advanced-age patients or those with severe cardiopulmonary comorbidities are often poor candidates for this surgery due to limited tolerability.

Carotid Artery Stenting (CAS) via the transfemoral approach has emerged as a minimally invasive alternative to CEA, particularly suitable for high-risk surgical patients. Although CAS reduces perioperative trauma to some extent, the transfemoral route still requires navigation through the aortic arch and carotid tortuosity, elevating the risk of microemboli dislodgement and distal cerebral embolism (6, 7). This embolic risk becomes notably heightened in cases involving aortic arch atherosclerosis, complex anatomy, or severe vascular tortuosity (8).

Currently, neuroprotective strategies can be categorized into three primary mechanisms based on hemodynamic control: dynamic flow reversal, as utilized in the Transcarotid Artery Revascularization (TCAR) system (9–12); proximal endovascular occlusion device, exemplified by the MO.MA device (13); and distal filter protection under antegrade flow conditions (14). While flow reversal has gained widespread recognition through large-scale registries like ROADSTER, it may be limited by flow intolerance in some patients. Conversely, transcervical CAS (TC-CAS), the use of distal filter protection under antegrade flow conditions offers a familiar and effective alternative that bypasses the aortic arch while maintaining continuous cerebral perfusion. By combining the safety of direct carotid access with the precision of distal embolic capture, this approach specifically addresses the embolic risks in transfemoral navigation without the complexities of hemodynamic reversal. Despite its potential, real-world comparative data between TC-CAS, transfemoral CAS (TF-CAS), and CEA remain limited. Therefore, this study aims to evaluate our single-center experience with this specific technique.

## Methods

### Study design and setting

This was a single-center, retrospective study conducted at The First Affiliated Hospital of Wenzhou Medical University. We reviewed consecutive patients who underwent carotid artery revascularization between January 2020 to December 2024. A total of 220 patients were included in the final analysis: 81 patients in the TF-CAS group, 50 patients in the TC-CAS group, and 89 patients in the CEA group. Research approval was obtained from the Ethics Committee of the First Affiliated Hospital of Wenzhou Medical University (No. KY2023-R231).

### Patient population

Patients with carotid artery stenosis who met the following inclusion criteria were enrolled in this study: (1) Symptomatic Carotid Stenosis: Patients with stenosis of the ipsilateral internal carotid artery measuring ≥ 50% based on doppler ultrasound, CTA, or MRA. These patients must have experienced one or more ipsilateral neurological events attributable to carotid stenosis within the past 6 months, including: Transient Ischemic Attack (TIA), Ischemic Stroke, Amaurosis fugax. (2) Asymptomatic Carotid Stenosis: Patients with severe stenosis of the ipsilateral internal carotid artery measuring ≥ 70% based on doppler ultrasound, CTA, or MRA. These patients must have no history of ipsilateral neurological symptoms (TIA, stroke, or amaurosis fugax) attributable to the stenosis at any point in time. The degree of carotid artery stenosis was measured according to NASCET. Neurological evaluations were performed by independent neurologists blinded to the treatment groups. Patients were assigned to one of the three treatment groups (TF-CAS, TC-CAS, or CEA). CEA was generally preferred for patients with low surgical risk and favorable neck anatomy. TF-CAS was typically chosen for patients deemed “high-risk” for open surgery but with favorable aortic arch and femoral access. TC-CAS was selectively employed for patients with “hostile” aortic arches or difficult transfemoral access, including Type III aortic arches, severe aortic arch atherosclerosis, carotid tortuosity, or significant peripheral artery disease that precluded safe femoral access. All patients received standardized medical therapy, including antiplatelet agents and statins, before undergoing any of the three procedures.

### Procedures

All procedures were performed by experienced vascular surgeons in carotid revascularization techniques.

#### Transcervical carotid artery stenting (TC-CAS)

Procedures were performed under general anesthesia. A small incision 2–3 cm in length was made at the base of the neck to expose the common carotid artery. Following heparinization, a 6-0 Prolene purse-string suture was placed on the anterior wall of common carotid artery, direct puncture was made and a 6-French sheath (Cordis, length 25 cm) was introduced into the common carotid artery on a 0.035 guidewire. Angiography was limited to the field of the target carotid artery. Cerebral protection was achieved using a distal filter protection device (Abbott, Embolishield NAV6^TM^) placed distal to the lesion. The stenosis was predilated, a self-expanding stent (primarily Abbott Xact, with other devices selected according to vessel diameter and anatomy) was deployed across the lesion, followed by post-dilation (if necessary). Finally, an angiography was performed to confirm there was no ≥ 30% residual stenosis and remove the distal filter protection device using a retrieval catheter. The purse-string was tied, and the incision closed.

#### Transfemoral carotid artery stenting (TF-CAS)

Procedures were performed under local anesthesia with conscious sedation. Access was obtained via the common femoral artery using standard Seldinger technique. Following systemic heparinization, the angiographies of the aortic arch and its branches were performed. The target carotid artery was selectively catheterized. A 6-French sheath, with 90 cm length was introduced into the common carotid artery on a stiff Amplatz guidewire. The remainder of the procedures were carried out in the same manner as TC-CAS.

#### Carotid endarterectomy (CEA)

All of the procedures were typically performed under general anesthesia via an incision along the anterior border of the sternocleidomastoid muscle. Eversion CEA was performed in 82 cases. The common carotid artery, internal carotid artery, and external carotid artery were exposed and controlled. Following systemic heparinization, the internal carotid artery is obliquely transected at its origin and everted to allow circumferential removal of the atherosclerotic plaque. The incision of the common carotid artery is extended proximally 1–2 cm to ensure complete clearance. The artery is then reimplanted into the common carotid artery using end-to-end anastomosis. The other 7 cases received standard CEA with shunt because the monitored stump pressure was below 50 mmHg. The arteriotomy was closed either primarily or with a patch angioplasty.

### Follow-up

Patients were scheduled for clinical follow-up and duplex ultrasound surveillance at 1 month, 3 month, 6 months post-procedure. Follow-up data were collected through outpatient clinic visits or telephone contact.

### Statistical analysis

Statistical analysis was performed using SPSS version 27. Continuous variables are presented as mean ± standard deviation (SD) and were compared using one-way analysis of variance (ANOVA). Categorical variables are presented as numbers and percentages [n (%)] and were compared using the Chi-square test or Fisher's exact test, as appropriate. For non-normally distributed continuous variables, data are presented as median (interquartile range [IQR]) and compared using the Kruskal–Wallis test. Due to the low number of outcome events across all binary endpoints (events per variable [EPV] < 5), multivariable logistic regression was not performed as the primary analysis. Exploratory multivariable logistic regression analyses adjusting for smoking status and hypertension were performed for surgical complications (Supplementary Table S1) and perioperative stroke (Supplementary Table S2); however, given the very low EPV, these results should be interpreted with caution. A two-sided *P* value < 0.05 was considered statistically significant.

## Results

### Patient population and baseline characteristics

A total of 220 patients undergoing carotid revascularization were enrolled in this study, with 81 patients in the transfemoral carotid artery stenting (TF-CAS) group, 50 in the transcervical carotid artery stenting (TC-CAS) group, and 89 in the carotid endarterectomy (CEA) group. The baseline characteristics of the three groups are presented in Table 1. There were no statistically significant differences observed in age (TF-CAS: 71.1 ± 8.6 years, TC-CAS: 71.4 ± 7.6 years, CEA: 69.4 ± 7.7 years; *p* = 0.267), sex distribution (*p* = 0.837), BMI (*p* = 0.396), alcohol consumption (*p* = 0.256), diabetes mellitus (*p* = 0.375), coronary artery disease (*p* = 0.156), symptomatic status (*p* = 0.788), or history of stroke (*p* = 0.593). Among symptomatic patients, the median time from the index neurological event to revascularization was 30 days (IQR 10–75) for TF-CAS, 20 days (IQR 15–60) for TC-CAS, and 25 days (IQR 9.5–67.5) for CEA, with no significant difference among the three groups (Kruskal–Wallis test, *p* = 0.870). However, significant differences were found in smoking status (*p* = 0.010) and the prevalence of hypertension (*p* = 0.018) among the groups. Specifically, the TC-CAS group had a lower proportion of smokers (24.0%) compared to TF-CAS (50.6%) and CEA (43.8%), and a higher prevalence of hypertension (84.0%) compared to TF-CAS (61.7%) and CEA (74.2%).

**Table 1:** Baseline characteristics of patients in the TF-CAS, TC-CAS, and CEA groups.

Characteristic	TF-CAS (*n* = 81)	TC-CAS (*n* = 50)	CEA (*n* = 89)	*P* value
Age (years)	71.1 ± 8.6	71.4 ± 7.6	69.4 ± 7.7	0.267
Gender, *n* (%)				0.837
Male	68 (84.0%)	40 (80.0%)	74 (83.1%)	
Female	13 (16.0%)	10 (20.0%)	15 (16.9%)	
BMI	23.6 ± 2.6	23.1 ± 2.6	23.0 ± 3.6	0.396
Smoking, *n* (%)	41 (50.6%)	12 (24.0%)	39 (43.8%)	0.01
Drinking, *n* (%)	29 (35.8%)	12 (24.0%)	33 (37.1%)	0.256
Diabetes mellitus, *n* (%)	32 (39.5%)	26 (52.0%)	40 (44.9%)	0.375
Hypertension, *n* (%)	50 (61.7%)	42 (84.0%)	66 (74.2%)	0.018
Coronary artery disease, *n* (%)	15 (18.5%)	9 (18.0%)	8 (9.0%)	0.156
Symptomatic status, *n* (%)				0.788
Symptomatic	13 (16.0%)	7 (14.0%)	11 (12.4%)	
Asymptomatic	68 (84.0%)	43 (86.0%)	78 (87.6%)	
History of stroke, *n* (%)	10 (12.3%)	9 (18.0%)	11 (12.4%)	0.593
Peripheral artery disease, *n* (%)	2 (2.5%)	9 (18.0%)	5 (5.6%)	0.003
Supra-aortic anatomy, *n* (%)				
Type III aortic arch	1 (1.2%)	13 (26.0%)	7 (7.9%)	<0.001
Bovine arch	1 (1.2%)	4 (8.0%)	4 (4.5%)	0.160
Aortic arch ulcer	0 (0.0%)	4 (8.0%)	3 (3.4%)	0.040
Carotid tortuosity	3 (3.7%)	8 (16.0%)	6 (6.7%)	0.034
Severe calcification	3 (3.7%)	7 (14.0%)	8 (9.0%)	0.106
Mixed plaque	2 (2.5%)	3 (6.0%)	7 (7.9%)	0.296

Data are presented as mean ± standard deviation or number (%).

### Perioperative outcomes

The perioperative outcomes for all 220 patients are summarized in Table 2. Overall perioperative stroke rates were low and showed no significant difference among the three groups (*P* = 0.802). An exploratory multivariable logistic regression adjusting for smoking and hypertension confirmed no significant association between procedure type and perioperative stroke (*p* = 0.779; Supplementary Table S2); however, the extremely wide confidence intervals reflect the very low event count and these results should be interpreted as exploratory. Surgical complications occurred in 0%, 4.0%, and 7.9% of TF-CAS, TC-CAS, and CEA patients, respectively (*P* = 0.035). While hematoma rates were similar across cohorts (*P* = 0.219), cranial nerve injury occurred exclusively in the CEA group (5.6%, *n* = 5; *P* = 0.023). An exploratory multivariable analysis for surgical complications is provided in Supplementary Table S1, but should be interpreted with caution given the complete separation in the TF-CAS group (zero events). No perioperative myocardial infarction or death occurred in any group. Hospital costs were significantly lower in the CEA group (25.0 ± 9.8 × 10^3^ CNY) compared to TF-CAS (57.6 ± 22.8 × 10^3^ CNY) and TC-CAS (56.4 ± 20.1 × 10^3^ CNY) (*p* < 0.001). Operative time was shortest in the TF-CAS group (73.2 ± 26.9 min), followed by TC-CAS (98.4 ± 21.6 min), and longest in the CEA group (112.1 ± 33.0 min) (*p* < 0.001). Additionally, among the subset of patients who underwent perioperative DW-MRI, the incidence of new lesions was TF-CAS 38.5% (5/13), TC-CAS 25.6% (10/39), and CEA 24.0% (6/25). Due to the significant selection bias in screening protocols, these MRI data are presented as descriptive observations rather than systematic comparative evidence.

**Table 2:** Comparison of perioperative outcomes among the TF-CAS, TC-CAS, and CEA groups.

Outcome	TF-CAS (*n* = 81)	TC-CAS (*n* = 50)	CEA (*n* = 89)	*P* value
Surgical complications, *n* (%)	0 (0%)	2 (4.0%)	7 (7.9%)	0.035
Hematoma	0 (0%)	2 (4.0%)	3 (3.4%)	0.219
Cranial nerve injury	0 (0%)	0 (0%)	5 (5.6%)	0.023
Perioperative stroke rate, *n* (%)	2 (2.5%)	1 (2.0%)	1 (1.1%)	0.802
Mild (Reversible)	1 (1.2%)	1 (2.0%)	1 (1.1%)	0.905
Severe (Irreversible)	1 (1.2%)	0 (0%)	0 (0%)	0.433
Perioperative MI/death, *n* (%)	0 (0%)	0 (0%)	0 (0%)	1
Hospital stay (days)	10.8 ± 4.3	10.8 ± 3.2	13.6 ± 4.5	< 0.001
Hospital cost (10^3^ CNY)	57.6 ± 22.8	56.4 ± 20.1	25.0 ± 9.8	< 0.001
Operative time (minutes)	73.2 ± 26.9	98.4 ± 21.6	112.1 ± 33.0	< 0.001
Preoperative mRS				0.233
mRS ≤ 2	80 (98.8%)	49 (98.0%)	84 (94.4%)	
mRS > 2	1 (1.2%)	1 (2.0%)	5 (5.6%)	
Postoperative mRS				0.429
mRS ≤ 2	79 (97.5%)	49 (98.0%)	84 (94.4%)	
mRS > 2	2 (2.5%)	1 (2.0%)	5 (5.6%)	
Patients with perioperative MRI, *n* (%)	13 (16.0%)	39 (78.0%)	25 (28.1%)	
New lesions on MRI, *n* (%)^*^	5 (38.5%)	10 (25.6%)	6 (24.0%)	

Exploratory multivariable logistic regression was performed adjusting for smoking status and hypertension (Supplementary Tables S1 and S2). Due to the low number of events (EPV < 5 for all binary endpoints), these analyses are subject to a high risk of overfitting and should be interpreted with caution.

* Percent of patients who underwent perioperative MRI (TF-CAS N = 13, TC-CAS N = 39, CEA N = 25).

### Six-month follow-up outcomes

Follow-up results at six months are presented in Table 3. The incidence of restenosis at 6 months did not show a significant difference among the groups (TF-CAS: 3.7%, *n* = 3; TC-CAS: 2.0%, *n* = 1; CEA: 4.5%, *n* = 4; *p* = 0.752). Similarly, the occurrence of stroke/TIA during the six-month follow-up period was not significantly different (*p* = 0.477), with one case occurring in the CEA group (1.1%, *n* = 1) and none in the TF-CAS or TC-CAS groups. There were no cases of myocardial infarction, death, or aneurysm reported in any of the groups during the six-month follow-up.

**Table 3:** Six-month follow-up clinical outcomes of patients in the TF-CAS, TC-CAS, and CEA groups.

Outcome	TF-CAS (*n* = 81)	TC-CAS (*n* = 50)	CEA (*n* = 89)	*P* value
Restenosis, *n* (%)	3 (3.7%)	1 (2.0%)	4 (4.5%)	0.752
Stroke/TIA, *n* (%)	0 (0%)	0 (0%)	1 (1.1%)	0.477
Myocardial infarction/death, *n* (%)	0 (0%)	0 (0%)	0 (0%)	1
Aneurysm, *n* (%)	0 (0%)	0 (0%)	0 (0%)	1

## Discussion

Carotid endarterectomy (CEA) remains the standard procedure for treating carotid artery stenosis. Transfemoral carotid artery stenting (TF-CAS) has evolved as an option for patients with high surgical risk factors; however, TF-CAS has been limited by the relatively higher peri-procedural stroke rates than CEA. Transcervical artery stenting (TC-CAS) represents a newer endovascular technique designed to mitigate some limitations associated with traditional TF-CAS, particularly related to aortic arch manipulation and distal embolic protection in high-risk anatomies. This study provides a valuable direct comparison of TC-CAS with both TF-CAS and CEA based on real-world data from a single center, offering insights into its safety, efficacy, and procedural characteristics.

A primary concern in carotid revascularization is periprocedural stroke. Our study demonstrates remarkably low and comparable overall perioperative stroke rates across all three groups (TF-CAS 2.5%, TC-CAS 2.0%, CEA 1.1%; *p* = 0.802). An exploratory multivariable logistic regression adjusting for smoking and hypertension confirmed that this finding was consistent. This finding is consistent with the pooled results of major randomized controlled trials like CREST and ACST-2 which showed similar periprocedural stroke and death rates between CAS and CEA in selected populations (15–17). The low stroke rate observed in the TC-CAS group is particularly encouraging. A key advantage of TC-CAS over TF-CAS when using distal filters is the significantly shorter working distance. This reduced distance facilitates more stable and precise deployment and retrieval of the protection device, potentially minimizing the risk of filter dislodgement or microemboli peri-filter leakage, which can occur during longer-distance manipulation with transfemoral access. Furthermore, TC-CAS is specifically designed to bypass complex or diseased aortic arches, bovine anatomy, or severe iliofemoral peripheral artery disease, which can increase the risk of stroke and procedural difficulty with TF-CAS (18–20). The favorable perioperative stroke outcomes in our TC-CAS cohort align with findings from large perioperative TCAR registries, such as ROADSTER and ROADSTER 2, which reported low periprocedural stroke rates, including in patients deemed high-risk for TF-CAS or CEA (10, 11). However, it is critical to distinguish the neuroprotection mechanisms between these approaches. In our study, TC-CAS was performed using a distal filter protection device (Abbott Embolishield NAV6™) placed distal to the lesion to capture potential embolic debris. In contrast, the TCAR system utilized a dynamic flow reversal system, which temporarily reverses carotid blood flow to prevent emboli from reaching the cerebral circulation. While both techniques aim to mitigate the risk of stroke associated with aortic arch manipulation—a major limitation of TF-CAS—the use of distal filters avoids flow reversal-related intolerance but may differ in its effectiveness against microembolization.

Beyond stroke, the overall surgical complication rate differed significantly among groups (*P* = 0.035), driven primarily by cranial nerve injury occurring exclusively in the CEA group (5.6%, *n* = 5; *P* = 0.023). No surgical complications were observed in the TF-CAS group, and TC-CAS had a low complication rate of 4.0%. An exploratory multivariable analysis (Supplementary Table S1) adjusting for smoking and hypertension yielded a non-significant result for three groups.These findings suggest that TC-CAS is a safe alternative, particularly for patients who may be unsuitable for CEA due to prior surgery or radiation (21). Hematoma rates were low and comparable across all groups, suggesting that the direct carotid access site for TC-CAS, when performed appropriately, does not appear to introduce a significantly higher risk of access-site hematoma requiring intervention compared to the femoral access for TF-CAS or the neck incision for CEA (22).

Our 6-month follow-up data indicate that TC-CAS provides short-term durability and clinical efficacy comparable to TF-CAS and CEA. Restenosis rates at 6 months were low and not significantly different between the groups (TC-CAS 2.0% vs. TF-CAS 3.7% vs. CEA 4.5%; *p* = 0.752). Major adverse cardiac and cerebrovascular events (stroke/TIA, MI/death, aneurysm) were also low and similar across all groups, with no events in the TC-CAS group. While 6-month follow-up is relatively short, these early results suggest that TC-CAS outcomes are not inferior to established methods in the immediate post-procedural and short-term period (23).

Despite the valuable insights, our study has limitations common to single-center, non-randomized designs, including potential selection bias and limited generalizability. The sample size, especially for the TC-CAS group, may limit the power to detect rarer complications or subtle outcome differences. The relatively short 6-month follow-up is another limitation, precluding conclusions about long-term durability. Furthermore, the low number of outcome events (EPV < 5 for all binary endpoints) precluded reliable multivariable logistic regression as a primary analysis. The exploratory multivariable analyses provided in Supplementary Tables S1 and S2 are subject to a high risk of overfitting, and the extremely wide confidence intervals should be interpreted with considerable caution. Lastly, the differing rates of perioperative MRI make direct comparison of silent cerebral embolization challenging across all patients.

In conclusion, this study suggests that transcervical artery stenting (TC-CAS) is a promising and feasible technique for carotid revascularization, demonstrating favorable perioperative safety and short-term clinical outcomes comparable to TF-CAS and CEA. TC-CAS offers distinct perioperative advantages, including the avoidance of cranial nerve injury compared to CEA and potentially improved embolic protection over TF-CAS in patients with challenging anatomy. While hospital costs are higher, TC-CAS represents a promising evolution in carotid stenting, providing a valuable alternative, particularly for patients less suitable for TF-CAS or CEA. Future multi-center studies with longer-term follow-up are warranted to further define the optimal role of TC-CAS in the carotid revascularization landscape.

## Data Availability

The raw data supporting the conclusions of this article will be made available by the authors, without undue reservation.
