# Supplementary material for: Transcervical carotid artery stenting compared to transfemoral carotid artery stenting and carotid endarterectomy: perioperative and short-term results from a single center
Source: Front Cardiovasc Med. 2026 Jun 3;13:1743275. doi: 10.3389/fcvm.2026.1743275 (PMC13271929; doi:10.3389/fcvm.2026.1743275)
Supplement: Supplementary file 1 [file Table1.docx]

| Table S1. Exploratory multivariable logistic regression analysis for surgical complications | | | |
| --- | --- | --- | --- |
| Variable | Odds Ratio | 95% confidence interval | P-value |
| Smoking | 1.292 | 0.299-5.584 | 0.731 |
| Hypertension | 0.509 | 0.118-2.2 | 0.366 |
| Procedure |  |  |  |
| CEA | / | / | 0.692 |
| TF-CAS | 6.65×10^-9^ | / | 0.997 |
| TC-CAS | 0.486 | 0.094-2.519 | 0.39 |

*CEA, carotid endarterectomy (reference group); TF-CAS, transfemoral carotid artery stenting; TC-CAS, transcervical carotid artery stenting. The model was adjusted for smoking status and hypertension. / Indicates that the odds ratio or confidence interval could not be estimated due to complete separation (zero surgical complication events in the TF-CAS group). Given the low number of events (9 total surgical complications), these results should be interpreted as exploratory only.*
