# Supplementary material for: Transcervical carotid artery stenting compared to transfemoral carotid artery stenting and carotid endarterectomy: perioperative and short-term results from a single center
Source: Front Cardiovasc Med. 2026 Jun 3;13:1743275. doi: 10.3389/fcvm.2026.1743275 (PMC13271929; doi:10.3389/fcvm.2026.1743275)
Supplement: Supplementary file 2 [file Table2.docx]

| Table S2. Exploratory multivariable logistic regression analysis for perioperative stroke | | | |
| --- | --- | --- | --- |
| Variable | Odds Ratio | 95% confidence interval | P-value |
| Smoking | 2.268 | 0.221-23.31 | 0.491 |
| Hypertension | 0.856 | 0.084-8.774 | 0.896 |
| Procedure |  |  |  |
| CEA | / | / | 0.779 |
| TF-CAS | 2.38 | 0.209-27.098 | 0.484 |
| TC-CAS | 1.546 | 0.092-25.855 | 0.762 |

*CEA, carotid endarterectomy (reference group); TF-CAS, transfemoral carotid artery stenting; TC-CAS, transcervical carotid artery stenting. The model was adjusted for smoking status and hypertension. Given the very low event count (4 perioperative strokes), confidence intervals are extremely wide and these results should be interpreted as exploratory only.*
